# Undifferentiated embryonal liver sarcoma in childhood: A case report

**DOI:** 10.3892/ol.2014.2262

**Published:** 2014-06-18

**Authors:** BIN HE, KEYU XU, KATE HUANG, YUEHAN HUANG, PENG LI, ZHENKUN CHEN, HONGQI SHI, QIYU ZHANG, YUNFENG SHAN

**Affiliations:** 1 Department of Hepatobiliary Surgery, The First Affiliated Hospital of Wenzhou Medical University, Wenzhou, Zhejiang 325000, P.R. China; 2 Department of Pathology, The First Affiliated Hospital of Wenzhou Medical University, Wenzhou, Zhejiang 325000, P.R. China

**Keywords:** undifferentiated embryonal sarcoma, liver, children, treatment

## Abstract

In order to improve the diagnosis and therapy of undifferentiated embryonal liver sarcoma (UELS), the present study presents the case of a 9-year-old female with UELS and discusses UELS in childhood. The patient presented with abdominal pain and fever. The laboratory tests, radiographic examination and pathological features presented by the female were similar to those of typical cases of UELS reported in childhood. The patient initially received surgical treatment and the immunohistochemical findings suggested that the patient had UELS. The patient’s parents refused adjuvant chemotherapy and demonstrated a right prerenal mass 6 months post-surgery. Microscopic examination revealed that the tumor was evidence of undifferentiated embryonal sarcoma recurrence. However, the patient was comfortable and physical examination revealed no abnormal conditions. In addition, the laboratory results were normal. Abdominal computed tomography scan and ultrasound were performed every 3 months to monitor the tumor recurrence. At the time of writing, it has been 6 months after the second surgical procedure and there has been no appearence of abnormalities. Previous studies have shown that patients who receive combined therapy with complete tumor resection and adjuvant chemotherapy have a longer survival time than those who undergo surgical therapy alone. Complete tumor resection combined with adjuvant chemotherapy may reduce the risk of recurrence and enhance the survival time in patients with UELS.

## Introduction

Undifferentiated embryonal liver sarcoma (UELS) is a rare and highly malignant hepatic tumor of mesenchymal origin, which often occurs in individuals between 5 and 10 years old, as well as in young adults (1–3). Patients with UELS usually present with a painful right upper quadrant mass, fever and other symptoms, including weight loss, vomiting, nausea, anorexia and jaundice (4–6). Ultrasonography (US), computed tomography (CT), magnetic resonance imaging (MRI) and biopsy are used as diagnostic tools to identify other liver tumors. However, the typical radiological findings of liver tumors are also observed in other liver diseases, including cystic hydatid disease (5). Previous studies have shown that complete tumor resection followed by adjuvant chemotherapy and/or radiation improves survival and reduces recurrence in patients with UELS, and this has now become a current standard therapy for UELS (7–9). The present study presents a childhood case of recurrent UELS that was treated with surgical resection without chemotherapy, and discusses the clinical characteristics, laboratory test results, immunohistochemical findings and treatment for this rare disease. Written informed consent was obtained from the parents of the patient.

## Case report

### Clinical characteristics, laboratory findings and imaging

**Figure 1:**
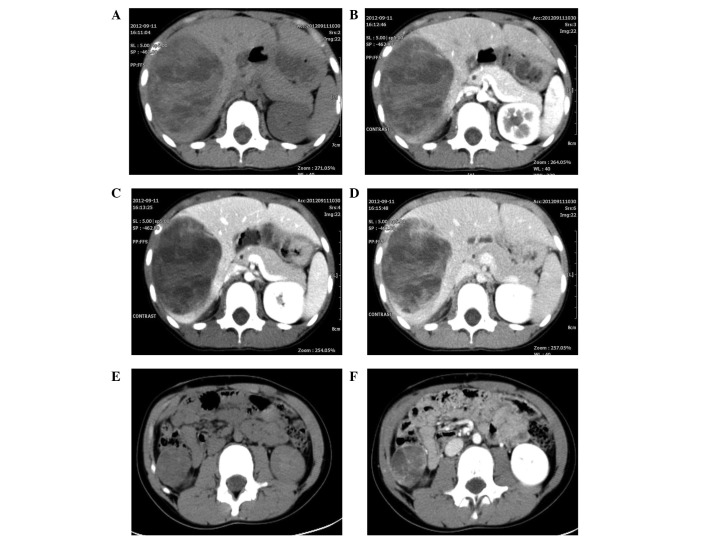
(A) Pre-contrast CT scan showing a mass with a well-defined border in the right lobe of the liver. (B–D) Following the administration of a contrast agent, CT scan reveals that the mass is marginally and heterogeneously enhanced. (E) Unenhanced CT reveals a right prerenal mass of ~2.7×2.4 cm^2^ in size with a well-defined border and low-density. (F) The density is increased marginally and heterogeneously with enhanced CT. CT, computed tomography.

A 9-year-old, previously healthy female was admitted to The First Affiliated Hospital of Wenzhou Medical University (Wenzhou, China), and presented with abdominal pain and fever. Upon examination, the patient’s temperature was 38°C, the pulse was 86 beats per minute, the blood pressure was 103/53 mmHg and the respiratory rate was 20 breaths per minute. Abdominal tenderness was identified in the right upper quadrant without rebound tenderness. The remainder of the examination was normal and the results are shown in Table I. Abdominal CT scan revealed a cystic and solid mass with a size of 11.5×8.3×6.7 cm^3^ and a well-defined border, which involved the right lobe of the liver and had a non-homogeneous density (Fig. 1A). The density was marginally and heterogeneously increased in the enhanced CT scan (Fig. 1B–D). The clinical impression was that this mass represented a hepatoblastoma.

### Treatment

Surgery was performed to resect the tumor. A right subcostal incision was made and, following entry into the abdomen, the ligamentum teres hepatis and falciform were incised. Examination of the future liver remnant and abdominal cavity revealed no evidence of tumor metastasis. The tumor had ruptured at the diaphragmatic surface and had adhered to the diaphragm. The surgical margin distance from the tumor was at ≥1 cm. The tumor was resected following the margins, until it was completely removed. A drainage tube was inserted under the right diaphragm and exited the body through the right abdominal wall. The abdominal wall was closed using standard procedures. During surgery, the patient had 300 ml bleeding and was transfused with 1.5 units of red blood cells.

### Pathologic findings

**Figure 2:**
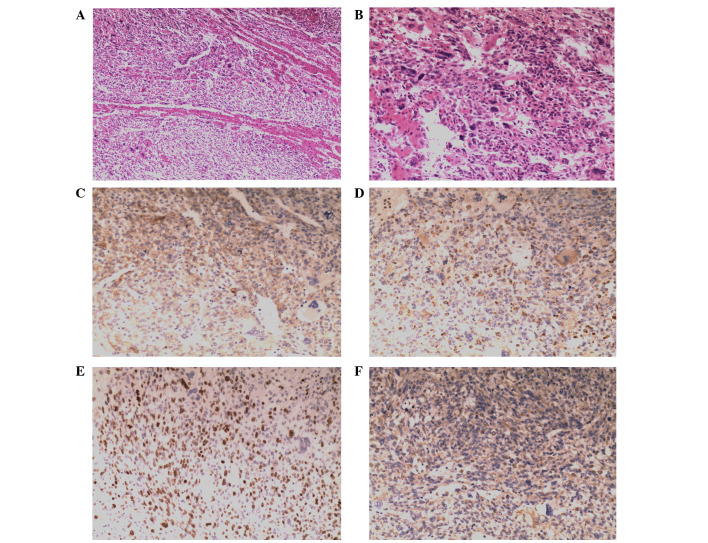
(A) Histological examination shows residual hepatocytes in the tumor (H&E stain; magnification, ×40) and (B) pleomorphically shaped tumor cells (H&E stain; magnification, ×100). Immunohistochemical staining (magnification, ×100) shows positive expression of (C) caldesmon, (D) cluster of differentiation 68, (E) Ki67 and (F) vimentin. H&E, hematoxylin and eosin.

Histological examination revealed pleomorphically shaped tumor cells with marked nuclei and clear to the eosinophilic cytoplasm (Fig. 2A–B). All the primary antibodies used in the present study are shown in Table II. Immunohistochemical staining of the tumor cells showed positive expression of caldesmon (Fig. 2C), cluster of differentiation (CD) 68 (Fig. 2D), Ki67 (Fig. 2E) and vimentin (Fig. 2F), but negative expression of α-fetoprotein (AFP), creatine kinase (CK), desmin, hepatocyte, muscle specific actin (MSA), myogenic differentiation (myod) 1, myoglobin and smooth muscle actin (SMA). On the basis of these findings, the patient was diagnosed with UESL, which was confirmed by two surgical pathologists.

### Clinical course

The patient was recommended for further treatment, which was refused due to socioeconomic and psychological reasons. Thus, adjuvant chemotherapy was not initiated. Following complete tumor resection, the patient was regularly examined. Abdominal CT scans and US were performed every 3 months to monitor tumor recurrence. At the 6-month follow-up, the patient was surviving free of disease; however, at the 9-month follow-up, unenhanced CT revealed a right, low-density prerenal recurrent mass with a size of ~2.7×2.4 cm^2^ and a well-defined border (Fig. 1E). Enhanced CT showed a marginally and heterogeneously increased density (Fig. 1F). The patient did not accept any further treatment. Two months later, CT scan demonstrated that the mass had enlarged to ~3.3×3.4 cm^2^. A right prerenal dissemination of UESL was suspected. Based on the patient’s condition, a second surgical procedure was required.

Upon second admission, the patient was comfortable and physical examination revealed there was no abnormal condition. The laboratory results are shown in Table I. Complete prerenal tumor resection was performed. There was tumor hemorrhage inside. Microscopic analysis showed that the tumor was evidence of undifferentiated embryonal sarcoma recurrence. However, the patient was comfortable and physical examination revealed no abnormal conditions. In addition, the laboratory results were normal. Abdominal computed tomography scan and ultrasound were performed every 3 months to monitor the tumor recurrence. At the time of writing, it has been 6 months after the second surgical procedure and there has been no appearence of abnormalities.

## Discussion

UELS is a rare, aggressive neoplasm with a poor prognosis, which predominantly affects children without gender predilection (1). UELS normally occurs in childhood, but has also been reported in adults (7). UELS typically presents with various non-specific tumor-associated symptoms, including right upper abdominal pain, distention, mass, nausea, swelling, vomiting, fever, weight loss, fatigue and jaundice (3,10,11). Occasionally, patients also have shoulder pain (10,12). Furthermore, physical examination occasionally reveals right upper abdominal tenderness. Laboratory studies are non-specific and have identified that patients with UELS exhibit low albumin, elevated lactic dehydrogenase, anemia and abnormal liver function (4,13,14). Moreover, serum assays for tumor markers, including AFP, cancer antigen 199 and carcinoembryonic antigen yield normal results.

The typical radiological finding of UELS is a large mass in the right liver lobe and occasionally in the left liver lobe. Abdominal CT images show large (range, 10–30 cm in size), cystic, solitary and well-circumscribed masses with variable areas of necrosis and hemorrhage (15,16). On enhanced CT, during the phase of the filling of the hepatic portal vein, a tumor was observed (15). Abdominal US reveals large multilocular (17) or unilocular (18) cystic and solid liver masses. The cystic region exhibits a large, mixed and disorderly low level echo (3,19), and the solid areas of the mass demonstrate a mixture of high and low level echos (19). Fluorine-18 fluorodeoxyglucose positron emission tomography (F-18 FDG PET)/CT may become increasingly important for the examination and treatment of UELS. F-18 FDG PET/CT may be a feasible and valuable imaging modality for further analysis of distant metastasis, as UELS and its metastases have strong F-18 FDG uptake (20).

Microscopically, UELS is characterized by cellular proliferation of oval, spindle or stellate pleomorphic cells with poorly defined cell borders (2,3). Immunocytochemically, the indices of the present patient were as follows: Caldesmon(+), CD68(+), Ki67(+), vimentin(+), AFP(−), CK(−), desmin(−), hepatocyte(−), MSA(−), myod1(−), myoglobin(−) and SMA(−). The outcome was approximately the same as that described previously (2). As in the present case, certain cases of UELS are positive for CD68 (21,22) and vimentin (23), which implies a mesenchymal origin. Moreover, in the present case, positive expression was found for caldesmon (24–26), which suggests sarcous origin. The positive expression of the proliferation marker Ki67 suggests it is an aggressive tumor (27,28).

The prognosis of UELS is poor even if the tumor is completely resected. Previous reports have demonstrated that treatment with surgery and adjuvant chemotherapy is curative for UELS in certain cases (1,9,29). May *et al* (4) reported five pediatric patients with UELS who were treated with radical resection followed by adjuvant chemotherapy or radiation and were alive without evidence of recurrence at a median of 53 months (4). Kim *et al* (9) reported that five pediatric patients who received similar therapy had a comparable prognosis, and proposed that a combined therapy of surgery and chemotherapy improves prognosis (9). Liver transplantation may be a potential treatment option for pediatric patients with UELS if it is feasible. Liver transplantation has been reported in pediatric patients with UELS (12,30,31). One study reported the case of a 6-year-old male who received a liver transplant combined with chemotherapy and was alive 6.5 years following surgery (30). Furthermore, another study reported a male who has been in good health and disease-free for 5 years following treatment with chemotherapy, surgical resection and liver transplantation (12). However, reports of liver transplantation to treat UELS are limited.

In conclusion, complete tumor resection combined with adjuvant chemotherapy may reduce the risk of recurrence and improve the survival time in patients with UELS. Further investigations are required to identify more effective strategies for treating pediatric patients with UELS.

## Figures and Tables

**Table I:** Laboratory data from a pediatric patient with undifferentiated embryonal liver sarcoma.

Parameter	First admission	Second admission
Blood		
White cell count (per mm^3^)	14,200	7,880
Differential count (%)		
Neutrophils	75.9	54.8
Eosinophils	0.2	0.9
Band forms	0.2	0.4
Monocytes	9.4	7.9
Lymphocytes	14.2	36
Erythrocyte count (per mm^3^)	3,860,000	4,770,000
Hemoglobin (g/dl)	11.5	14.3
Hematocrit (%)	33.8	41.4
Platelet count (per mm^3^)	323,000	192,000
Prothrombin time (sec)	14.3	13.8
Activated partial thromboplastin time (sec)	41.8	53.9
Serum AFP level (μg/l)	1.3	1.93
Serum CA-125 level (μU/ml)	30.7	-
Serum CA-19-9 level (U/ml)	-	5.0
Serum CEA level (μg/l)	0.8	1.2
Total bilirubin (μmol/l)	7	7
Direct bilirubin (μmol/l)	3	4
Indirect bilirubin (μmol/l)	4	3
Total protein (g/l)	59.6	67.1
Albumin (g/l)	32.1	41.0
Alanine aminotransferase (U/l)	38	10
Aspartate aminotransferase (U/l)	45	24
Alkaline phosphatase (U/l)	208	296
γ-glutamyl transferase (U/l)	50	10
Lactate dehydrogenase (U/l)	377	291
Creatine kinase (U/l)	20	144
Glucose (mmol/l)	5.2	5.0
Sodium (mmol/l)	138	137
Potassium (mmol/l)	4.25	3.97
Chloride (mmol/l)	101	104
Urea nitrogen (mmol/l)	7.0	3.4
Creatinine (μmol/l)	40	40
Uric acid (μmol/l)	177	307

AFP, α-fetoprotein; CA, cancer antigen; CEA, carcinoembryonic antigen.

**Table II:** Primary antibodies used in the present study.

Antibody	Clone	Source	Dilution
AFP (M)	ZSA06	Zhongshan Gold Bridge Biological Technology Co. (Beijing, China)	1:100
Caldesmon (M)	EP19	Zhongshan Gold Bridge Biological Technology Co. (Beijing, China)	1:100
CD68 (M)	KP1	Zhongshan Gold Bridge Biological Technology Co. (Beijing, China)	1:100
CK (M)	AE1	Zhongshan Gold Bridge Biological Technology Co. (Beijing, China)	1:100
Desmin (M)	ZC18	Zhongshan Gold Bridge Biological Technology Co. (Beijing, China)	1:100
Hepatocyte (M)	OCH1E5	Zhongshan Gold Bridge Biological Technology Co. (Beijing, China)	1:100
Ki67 (M)	K-2	Zhongshan Gold Bridge Biological Technology Co. (Beijing, China)	1:100
MSA (M)	HHF35	Zhongshan Gold Bridge Biological Technology Co. (Beijing, China)	1:100
Myodl (M)	5.2F	Zhongshan Gold Bridge Biological Technology Co. (Beijing, China)	1:100
Myoglobin (M)	Z001	Zhongshan Gold Bridge Biological Technology Co. (Beijing, China)	1:100
SMA (M)	IA4	Zhongshan Gold Bridge Biological Technology Co. (Beijing, China)	1:100
VIM (M)	V9	Zhongshan Gold Bridge Biological Technology Co. (Beijing, China)	1:100

## References

[1] Bisogno G, Pilz T, Perilongo G (2002). Undifferentiated sarcoma of the liver in childhood: a curable disease. Cancer.

[2] Wei ZG, Tang LF, Chen ZM, Tang HF, Li MJ (2008). Childhood undifferentiated embryonal liver sarcoma: clinical features and immunohistochemistry analysis. J Pediatr Surg.

[3] Li XW, Gong SJ, Song WH (2010). Undifferentiated liver embryonal sarcoma in adults: a report of four cases and literature review. World J Gastroenterol.

[4] May LT, Wang M, Albano E, Garrington T, Dishop M, Macy ME (2012). Undifferentiated sarcoma of the liver: a single institution experience using a uniform treatment approach. J Pediatr Hematol Oncol.

[5] Oral A, Yigiter M, Demirci E, Yildirim ZK, Kantarci M, Salman AB (2011). A case of undifferentiated embryonic liver sarcoma mimicking cystic hydatid disease in an endemic region of the world. J Pediatr Surg.

[6] Stocker JT, Ishak KG (1978). Undifferentiated (embryonal) sarcoma of the liver: report of 31 cases. Cancer.

[7] Lenze F, Birkfellner T, Lenz P (2008). Undifferentiated embryonal sarcoma of the liver in adults. Cancer.

[8] O’Sullivan MJ, Swanson PE, Knoll J, Taboada EM, Dehner LP (2001). Undifferentiated embryonal sarcoma with unusual features arising within mesenchymal hamartoma of the liver: report of a case and review of the literature. Pediatr Dev Pathol.

[9] Kim DY, Kim KH, Jung SE, Lee SC, Park KW, Kim WK (2002). Undifferentiated (embryonal) sarcoma of the liver: combination treatment by surgery and chemotherapy. J Pediatr Surg.

[10] Shehata BM, Gupta NA, Katzenstein HM (2011). Undifferentiated embryonal sarcoma of the liver is associated with mesenchymal hamartoma and multiple chromosomal abnormalities: a review of eleven cases. Pediatr Dev Pathol.

[11] Zaman S, Hanif G, Hussain M (2011). Hepatic tumours in childhood: an experience at the Children Hospital and Institute of Child Health, Lahore. J Pak Med Assoc.

[12] Kelly MJ, Martin L, Alonso M, Altura RA (2009). Liver transplant for relapsed undifferentiated embryonal sarcoma in a young child. J Pediatr Surg.

[13] Uchiyama M, Iwafuchi M, Yagi M (2001). Treatment of ruptured undifferentiated sarcoma of the liver in children: a report of two cases and review of the literature. J Hepatobiliary Pancreat Surg.

[14] Shattaf A, Jamil A, Khanani MF (2012). Undifferentiated sarcoma of the liver: a rare pediatric tumor. Ann Saudi Med.

[15] Yu RS, Chen Y, Jiang B, Wang LH, Xu XF (2008). Primary hepatic sarcomas: CT findings. Eur Radiol.

[16] Sakellaridis T, Panagiotou I, Georgantas T, Micros G, Rontogianni D, Antiochos C (2006). Undifferentiated embryonal sarcoma of the liver mimicking acute appendicitis. Case report and review of the literature. World J Surg Oncol.

[17] Joshi SW, Merchant NH, Jambhekar NA (1997). Primary multilocular cystic undifferentiated (embryonal) sarcoma of the liver in childhood resembling hydatid cyst of the liver. Br J Radiol.

[18] Chowdhary SK, Trehan A, Das A, Marwaha RK, Rao KL (2004). Undifferentiated embryonal sarcoma in children: beware of the solitary liver cyst. J Pediatr Surg.

[19] Gao J, Fei L, Li S (2013). Undifferentiated embryonal sarcoma of the liver in a child: A case report and review of the literature. Oncol Lett.

[20] Lee MK, Kwon CG, Hwang KH (2009). F-18 FDG PET/CT findings in a case of undifferentiated embryonal sarcoma of the liver with lung and adrenal gland metastasis in a child. Clin Nucl Med.

[21] Nishio J, Iwasaki H, Sakashita N (2003). Undifferentiated (embryonal) sarcoma of the liver in middle-aged adults: smooth muscle differentiation determined by immunohistochemistry and electron microscopy. Hum Pathol.

[22] Scudiere JR, Jakate S (2006). A 51-year-old woman with a liver mass. Undifferentiated embryonal sarcoma of the liver. Arch Pathol Lab Med.

[23] Zheng JM, Tao X, Xu AM, Chen XF, Wu MC, Zhang SH (2007). Primary and recurrent embryonal sarcoma of the liver: clinicopathological and immunohistochemical analysis. Histopathology.

[24] Watanabe K, Tajino T, Sekiguchi M, Suzuki T (2000). h-Caldesmon as a specific marker for smooth muscle tumors. Comparison with other smooth muscle markers in bone tumors. Am J Clin Pathol.

[25] Nakayama H, Kamiji I, Naruse K (1998). Well differentiated adult-type fibrosarcoma arising from the occipital subcutaneous tissue in a 17-year-old man: case report with immunohistochemical study. Jpn J Clin Oncol.

[26] Nucci MR, O’Connell JT, Huettner PC, Cviko A, Sun D, Quade BJ (2001). h-Caldesmon expression effectively distinguishes endometrial stromal tumors from uterine smooth muscle tumors. Am J Surg Pathol.

[27] Settakorn J, Kaewpila N, Burns GF, Leong AS (2005). FAT, E-cadherin, beta catenin, HER 2/neu, Ki67 immuno-expression, and histological grade in intrahepatic cholangiocarcinoma. J Clin Pathol.

[28] Stroescu C, Dragnea A, Ivanov B (2008). Expression of p53, Bcl-2, VEGF, Ki67 and PCNA and prognostic significance in hepatocellular carcinoma. J Gastrointestin Liver Dis.

[29] Weitz J, Klimstra DS, Cymes K (2007). Management of primary liver sarcomas. Cancer.

[30] Dower NA, Smith LJ (2000). Liver transplantation for malignant liver tumors in children. Med Pediatr Oncol.

[31] Okajima H, Ohya Y, Lee KJ (2009). Management of undifferentiated sarcoma of the liver including living donor liver transplantation as a backup procedure. J Pediatr Surg.

